# Effectiveness of financial incentives for recruitment and retention of skilled health professionals for the public health system in Orissa, India

**DOI:** 10.1186/1753-6561-6-S5-O2

**Published:** 2012-09-28

**Authors:** Indira Chakravarthi

**Affiliations:** 1 Public Health Resource Network, New Delhi, India

## Introduction

Lack of skilled personnel for healthcare facilities in rural areas in India has been a chronic problem for several decades now. Since the 1990s, various measures have been recommended and adopted for recruitment and retention of doctors and other skilled health care personnel in rural areas. Providing financial incentives is reported to be the most common strategy. However, comprehensive evaluations of the effectiveness of these measures in the Indian context are rare. This study attempts to fill this gap by undertaking an assessment of the financial incentives scheme for the recruitment and retention of health professionals in the state of Orissa.

## Methods

This study is based on document reviews and open ended interviews conducted with doctors and other health officials at the state and district level. The districts, the health facilities and the doctors were purposively selected. Two districts from the 11 incentive districts (Koraput and Kalahandi) and one from the non-incentive districts (Mayurbhanj) were studied.

A total of 27 health facilities were selected. We interviewed a total number of 35 respondents. Of these, 30 doctors were from peripheral facilities of which 11 were from the non-incentive districts and 19 from the two incentive districts. The interview guide covered topics on the respondents’ views on the incentives scheme, reasons for joining Government health services, general unwillingness to work in remote areas and suggestions on attracting and retaining doctors in the government health services. Field work was conducted in December 2011-February 2012.

## Results

The results of the study indicate that incentives have not been very effective in improving availability of doctors in public health facilities, specifically in the peripheral institutions. It was not the monetary incentive that was the motivating factor for joining or staying in the public sector but a variety of personal and other reasons that influenced this choice. These reasons included limited options of getting jobs in private sector, provisions of reservation of seats for post-graduation for in-service doctors and unsatisfactory service and working conditions. Other factors like increasing administrative work load, poor infrastructure and overall backwardness of the region also influenced the choice to work in rural areas.

Vacancies in several non-incentive districts were equal to or even higher than that in the incentivized districts. The basis for selecting regions for which incentives are given was not very clear. The financial incentive measure remains a `temporary’ one, subject to annual renewal on concurrence from the Department of Finance. Some improvements due to the National Rural Health Mission seem to be at the cost of increasing administrative work of medical officers, leaving little time for clinical work and study.

## Discussion

While the shortage of doctors may be particularly acute in parts of Orissa, evidence from other parts of India shows that it afflicts all the states, to a greater or lesser extent. Further, the shortage is not limited to just rural facilities; public hospitals in urban areas too are facing similar problems. Reinforcing available evidence, our study shows that isolated measures focusing only on incentivization cannot redress the problem of chronic shortage of human resources in public health facilities in both rural and urban areas. Improving infrastructure, living and service conditions of the doctors along with incentives would go a long way in attracting and retaining doctors in public health facilities, specifically in rural areas.

Findings from our study also suggest the need for more research evidence to understand the persistent dysfunctional status of the public health facilities in the context of the role of the state and its political and economic ideologies. What are the barriers to achieving satisfactory working conditions for doctors, to health systems strengthening? Why only piece-meal measures from recommendations for comprehensive strategies and systemic approaches are implemented? Can the shortage of personnel for public sector health facilities be addressed without confronting the influence and impact of the larger political ideologies and compulsions of neo-liberalization, and the drive by the states towards promoting private interests in every sector, including healthcare? The answers to these will influence the very understanding of universal health coverage and strategies adopted for health systems strengthening.

## Competing interests

Author declares that she has no conflict of interest.

## Funding statement

The study was funded by the ICICI Foundation for Inclusive Growth-Centre for Child Health and Nutrition (IFIG-CCHN).

